# Synergistic Inhibition of PI3K and HSP90 Enhanced Antitumorigenic Efficacy in Adrenocortical Carcinoma

**DOI:** 10.21203/rs.3.rs-7761877/v1

**Published:** 2025-11-01

**Authors:** Prachi Mishra, Brieann Sobieski, Dipranjan Laha, Steven D. Forsythe, Min Shen, Ya-Qin Zhang, Mathew Hall, Bhavishya Ramamoorthy, Rob Grant, Nicholas L. Michael, Samira M. Sadowski, Jaydira del Rivero, Jonathan M. Hernandez, Naris Nilubol

**Affiliations:** 1 Surgical Oncology Program, National Cancer Institute, National Institutes of Health, Bethesda, MD, USA; 2 Chemical Genomics Center, National Center for Advancing Translational Sciences, National Institutes of Health, Rockville, MD, USA; 3 Developmental Therapeutics Branch, National Cancer Institute. National Institutes of Health, Bethesda, MD, USA

**Keywords:** Adrenocortical cancer, Heat shock protein 90, PI3K, combination therapy, quantitative high-throughput drug screening

## Abstract

Adrenocortical cancer (ACC) is a rare and aggressive malignancy with poor survival due to a lack of effective treatments; therefore, it is important to identify therapies to be readily studied in clinical trials. Quantitative high-throughput drug combination screening identified potent synergy between phosphatidylinositol-3-kinase (PI3K) inhibitor, PIK75 and heat shock protein 90 (HSP90) inhibitors, Ganetespib (STA9090), HSP990, or Luminespib (NVP-AUY922). Preclinical *in-vitro* and *in-vivo* studies were performed to validate the synergistic efficacy of the most effective HSP90 inhibitor and PI3K inhibitor combination in ACC cell lines, human ACC xenografts and patient-derived organoids (PDOs). Combination of PIK75 and STA9090, synergistically inhibited cell proliferation (monolayer and 3-dimensional), cell migration/invasion and epithelial-to-mesenchymal transition with decreased phosphorylated proteins in PI3K/mTOR signaling pathway. Due to the unavailability of PIK75 for clinical trial, another PI3K inhibitor, BGT226, which was clinically available and demonstrated a comparable synergistic efficacy with STA9090, was validated in the ACC cell lines. RNA sequencing analysis and phenotypic studies revealed that the BGT226-STA9090 combination induced autophagy-related cell death in ACC cells, unlike the PIK75-STA9090 combination which induced caspase-dependent apoptosis and G2/M cell cycle arrest. Further antitumor efficacy was confirmed by the BGT226-STA9090 combination in human ACC xenograft model and five PDOs with different pathogenic mutations. Conclusively, the combinations of PI3K and HSP90 inhibitors were highly effective in preclinical studies, warranting a clinical trial in patients with advanced ACC.

## INTRODUCTION

Adrenocortical cancer (ACC) is a rare (0.5–2 cases per million per year) and aggressive endocrine cancer with poor 5-year survival (<40%)^1^. The treatment of ACC remains challenging due to the lack of effective treatment; surgical removal of the tumor is the only curative treatment option in patients with early-stage disease. Despite a complete resection, the recurrence and metastasis rates remain high (50–80%)^2^. Mortality in ACC is commonly caused by progressive distant metastasis due to the lack of effective systemic treatment options or complications from uncontrolled hypercortisolism. To date, mitotane is the only drug approved by the FDA for advanced disease^3^. The current standard of care chemotherapy (etoposide, doxorubicin, and cisplatin) only provides short-term tumor control in 23% of patients^4^. Therefore, it is critically important to identify new effective treatments that can be readily studied in a clinical trial.

Drug development studies targeting molecular vulnerabilities in cancers from our group and others have unraveled robust responses from combinatorial targeted therapies in comparison to monotherapies^5,6^. A common disadvantage observed in a single-drug targeted therapy is drug resistance, which can be overcome by combining therapies to synergistically target multiple mechanisms in the tumor. Moreover, combination therapy may be more effective in highly heterogenous tumors; a characteristic frequently observed in ACC metastases^7^.

Heat shock protein 90 (HSP90) is a ubiquitous molecular chaperone that maintains the stability of proteins under physiological stress and plays an important role in tumorigenesis. A large number of HSP90 client proteins including protein kinases, transcription factors, and steroid hormone receptors^8^, have been involved in oncogenic processes such as metastasis, increased proliferation, invasion, angiogenesis, evasion of apoptosis, and metabolic reprogramming^9–11^.

Thus, it is a potentially good target to abrogate oncogenic signaling pathways. HSP90 inhibitors have demonstrated significant antitumor activities in numerous preclinical tumor models, in breast and lung cancer^12^. The combination of HSP90 inhibitors and chemotherapy had earlier shown clinical benefits in several phase I studies^13^. The second-generation synthetic inhibitors of HSP90, including Ganetespib (STA9090), Luminespib (NVP-AUY922), HSP990, and Onalespib have higher potency in comparison to the first-generation HSP90 inhibitors. These inhibitors achieved prolonged target inhibition, overcame drug resistance, decreased hepatotoxicity, and were observed to be well-tolerated in several clinical trials in patients with solid malignancies, non-small cell lung, colorectal, and breast cancer^14–16^. Understanding the molecular mechanisms of these inhibitors in combination with other drugs will positively maneuver future therapeutic studies in ACC.

Several well-known clients of HSP90 are members of the PI3K/AKT/mTOR pathway; protein kinases that have been targeted in multiple cancers including ACC. The PI3K/AKT/mTOR pathway members play a significant role in the intracellular signaling of several growth factors and are instrumental in the process of carcinogenesis, and are directly or indirectly linked to HSP90 to maintain their stability^17,18^. Although a monotherapy using pan-PI3K inhibitors, isoform-selective PI3K inhibitors, and dual PI3K/mTOR inhibitors showed promising anti-proliferative effects in several preclinical ACC models, we observed a lack of efficacy in clinical trials^19^. Thus, a combination therapy using HSP90 and PI3K inhibitors could be an effective strategy in ACC.

In this study, we performed quantitative high-throughput drug combination matrix screening in ACC cell lines. The most potent synergistic drug combinations from this screening were PIK75 (a selective inhibitor of the p110α subunit of PI3K) with one of the three second-generation HSP90 inhibitors (STA9090, HSP990, or NVP-AUY922). We validated the *in vitro* synergic antiproliferative effect between PIK75 and one of the three HSP90 inhibitors. Because PIK75 is not clinically available, we combined BGT226 (selective inhibitor of PI3K p110α subunit and mTOR) with STA9090 and found potent synergistic *in vitro and in vivo* antiproliferative effect in ACC cells and patient-derived organoids (PDOs). These combination treatments exhibited potent activity against ACC 3D-spheroids, colony formation, and cell migration and invasion at clinically achievable concentrations. We demonstrated the difference in mechanism of action between the PIK75 and HSP90 inhibitor combination, which induced G2M cell cycle arrest and caspase-dependent apoptosis, and the BGT226 and HSP90 inhibitor combination, which induced autophagic-related cell death without apoptosis. Taken together, the combinations of selective inhibitors of the PI3K p110α subunit and the second-generation HSP90 inhibitors should be studied in patients with advanced ACC.

## RESULTS

### Identification of HSP90 and PI3K inhibitors for combination therapy in adrenocortical carcinoma cells using a quantitative high-throughput drug screening

**Figure1.**
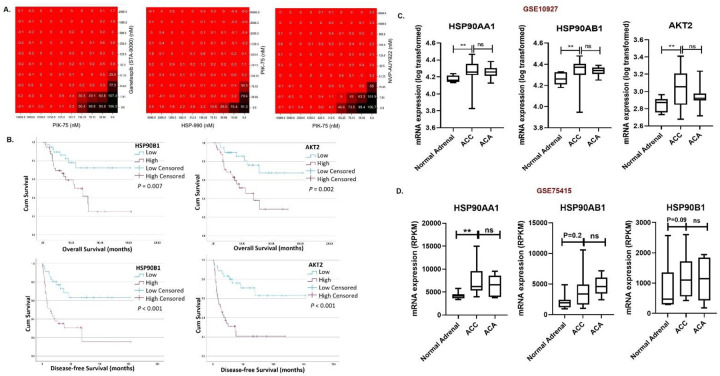
Candidate drug inhibitors and expression of their targets from HSP90 and PI3K pathway in adrenocortical carcinoma. A. Pairwise combinations of the test inhibitors using a wide range of concentrations (nM) in NCI-H295R cells, from low (right lower corner) to high (right upper and left lower corners), to measure the drugs synergy by using a 10×10 matrix for STA-9090 and PIK-75, HSP990 and PIK-75 and NVP-AUY922 and PIK-75. Black represents higher cell viability and red represents lower cell viability in percentage compared to controls. B. Kaplan Meyers plots showing ACC patients overall survival (OS) or disease-free survival (DFS) with high and low mRNA expression of *HSP90B1* (OS: *P* =0.007, DFS: *P* <0.001), and *AKT2* (OS: *P*=0.002, DFS: *P* <0.001). Graphs showing mRNA expression for key members of HSP90 (HSPAA1, HSP90AB1 and HSP90B1) and PI3K (AKT2, CDK4 and CDK1) family in GEO datasets C. GSE10927 Normal Adrenal (n=10), ACA (n= 22), ACC (n=33) and D. GSE75415; Normal Adrenal (n=7), ACA (n= 5), ACC (n=18). * *P*≤ 0.05, ** *P*≤ 0.01, *** *P*≤ 0.001. ACA; Adrenocortical Adenoma. ACC; Adrenocortical Carcinoma.

We previously conducted a quantitative high throughput screening (qHTS) using a pharmaceutical library comprising ~5,000 (either approved or investigational) drugs and the updated oncology-focused library^20,21^, to identify efficacious therapies for ACC cells. Among the most potent synergistic combinations from the database was one of the three HSP90 inhibitors (HSP990, NVP-AUY922, and STA-9090), with the PI3K inhibitor (PIK-75). These drug combinations were stringently selected from a primary list of 74 novel active drugs which were subjected to a secondary screening based on caspase 3/7 activity as an endpoint, along with corresponding cell viability assay. The top 30 candidate drugs with the highest antitumor activity were selected for further computerized 10×10 pairwise combination dose matrix analysis, to derive the most effective drug combinations for targeted therapy in ACC. A pairwise analysis of either STA-9090, HSP990, or NVP-AUY922 with PIK75 revealed synergistic efficacy, using the Bliss and High Single Agent (HSA) algorithms^22,23^, with a marked decrease in ACC cell viability at low and clinically achievable concentrations (Fig. 1a).

Next, we assessed whether primary human ACC samples had significantly differentially expressed genes of interest that their proteins are targeted by drug combinations using publicly available databases such as Gene Expression Omnibus (GEO) and The Cancer Genome Atlas (TCGA). This included mRNA expression of genes in HSP90 family (*HSP90AA1, HSP90AB1, HSP90B1, TRAP1*) and PIK3/AKT/mTOR pathway (*PIK3CA, PIK3CB, PIK3R1, AKT1, AKT2*) in TCGA (Supplementary Fig 1). In three of the GEO databases (GSE10927, GSE75415 and GSE12368), the cytosolic HSP90 (*HSP90B1* and/or *HSP90AA1* and *HSP90AB1*) and their clients belonging to the PI3K family (*AKT2*) were significantly overexpressed (*P* < 0.01) in the ACC tissues (Fig. 1b, 1d, Supplementary Fig. 2) compared to the normal adrenal and adrenocortical adenoma (ACA) tissue samples, irrespective of tumor stage. To assess the clinical relevance of the above genes, we analyzed the overall survival (OS) and disease-free survival (DFS) of patients with ACC in TCGA cohort (N=79) using mRNA expression of genes in the HSP90 and PI3K/AKT signaling pathway. We found that patients with ACC and high expression of *HSP90B1* (OS: *P* =0.007, DFS: *P* <0.001)*, HSP90B3P* (OS: *P* =0.092, DFS: *P* =0.084), *PIK3R1* (OS: *P* <0.010, DFS: *P* =0.092), *AKT2* (OS: *P*=0.002, DFS: *P* <0.001), *HSP90AB2P* (DFS: *P*=0.019) and *HSP90B2P* (DFS: *P*=0.044), were associated with either shorter OS or shorter DFS or both (Fig. 1c, Supplementary Fig. 3, 4). We found significantly higher *HSP90B1*, *HSP90B2P* and *AKT2* mRNA expression in primary human ACC samples with stage III/IV (N=31) and in samples with distant metastasis (N=15) (Supplementary Fig. 5). In addition, mRNA expression of *HSP90* family members in TCGA ACC cohort was statistically significantly and linearly correlated with *MKI67* (cell proliferative index)^24^ and *CTNNB1* (β-catenin)^25^, two well-known genes associated with poor prognosis in human ACC (Table 1). This observation suggested that ACC may be effectively targeted by the combination of PI3K inhibitor and HSP90 inhibitor.

### Synergistic antiproliferative activity of combined HSP90 and PI3K inhibitors in adrenocortical carcinoma cells

**Figure 2.**
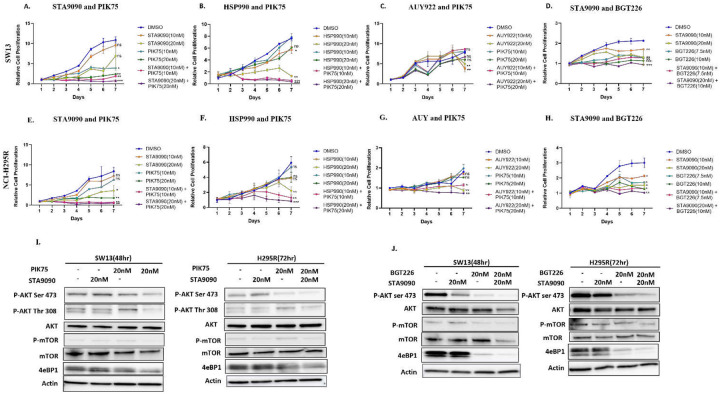
Significant reduction in proliferation of ACC cells after combined treatment of HSP90 and PI3K inhibitors. Graphs representing proliferation curves of SW13 cells treated with HSP90 and PI3K drugs either as monotherapy or combination therapy of A. STA9090 and PIK75 B. HSP990 and PIK75 C. AUY922 and PIK7 and D. STA9090 and BGT226 for 7 days. Graphs representing proliferation curves of NCI-H295R cells treated with HSP90 and PI3K drugs either as monotherapy or combination therapy of E. STA9090 and PIK75 F. HSP990 and PIK75 G. AUY922 and PIK7 and H. STA9090 and BGT226 for 7 days. Statistical significance was calculated for day 7 using student’s t-test. Western blots showing I. inhibition of the protein expression of the PI3K pathway members phospho-AKT (ser 473 and thr 308), phospho-mTOR and 4eBP1 after treatment of the inhibitors STA9090 and PIK75 for 48hrs and 72hrs respectively, and J. inhibition of phospho-AKT(ser 473), phospho-mTOR and 4eBP1 after treatment of the inhibitors STA9090 and BGT226 for 48hrs and 72hrs respectively.

To confirm if the synergistic efficacy of these HSP90 and PI3K inhibitor combinations obtained from the combination matrix are achievable clinically, we tested the anti-proliferative effect in a range of five different effective concentrations in both SW13 and NCI-H295R cells. A significant antiproliferative activity was observed with increasing doses of STA9090 and PIK75 in both the ACC cell lines in monotherapy or in combination (Fig. 2a and 2e). Furthermore, the combination of STA9090 and PIK75 showed a synergistic antiproliferative effect (CI<1) using the Chou-Talay method (Supplementary Fig. 6a). The synergy of STA9090 and PIK75 was more consistent across the selected concentrations as compared to HSP990 or AUY226 and PIK75 combination (Fig. 2a–c, 2e–g). Western blots confirmed that the combination treatment of STA9090 and PIK75 had significantly reduced the protein expression of the downstream PI3K pathway members phospho-AKT (ser473, or thr308), 4eBP1, and modestly phospho-mTOR (Fig. 2i).

Because PIK75 is not clinically available and we aimed to identify an effective combination therapy from our studies to develop a clinical trial in patients with advanced ACC, we tested a new clinical-trial-ready PI3K inhibitor, BGT226, which inhibits PI3K protein by binding to the α subunit at p110 (like PIK75) and the mTOR protein^26^. The single-drug treatment with BGT226 demonstrated a dose-dependent anti-proliferative efficacy (Fig. 2d, 2h) in NCI-H295R and SW13 cells. Additionally, combinations of BGT226 and either STA9090 or HSP990 showed a significant synergistic anti-proliferative effect (Fig. 2d, 2h, Supplementary Fig. 6b, 7a, 7b), however the combination of BGT226 and STA9090, demonstrated a synergistic anti-proliferative effect with a higher efficacy compared to the combination of BGT226 and HSP990. Moreover, the combination of STA9090 and BGT226 effectively decreased phospho-AKT (ser473, or thr308), phospho-mTOR, and 4eBP1 (Fig. 2j), suggesting that the efficacy is clinically achievable. We observed a reduction in the phosphorylated PI3K-pathway proteins (pAKT and pmTOR) in ACC cells treated with BGT226 and STA9090, compared to PIK75-STA9090 treated ACC cells.

### Combined HSP90 and PI3K inhibitors inhibited multicellular aggregates, induced apoptosis, and decreased cell migration and invasion

**Figure 3.**
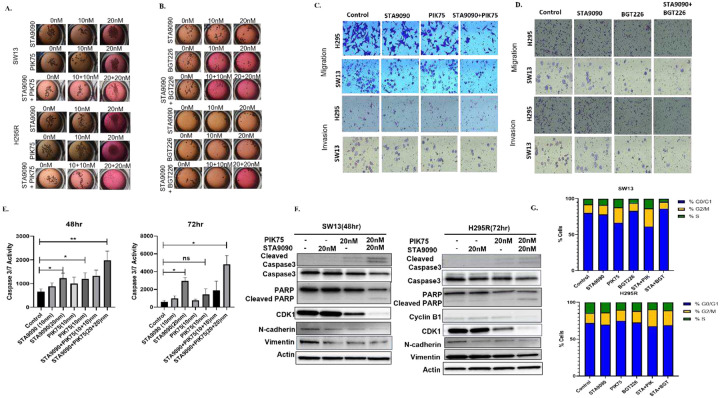
Increased anti-tumor activities by the combination treatment of HSP90 and PI3K inhibitors. Monotherapy and combination therapy of A. STA9090 and PIK75 and B. STA9090 and BGT226 demonstrating disintegration of 3-dimensional multicellular aggregates of cells. Inhibition of invasion and migration of cells observed after treatment of drugs either individually or in combination of C. STA9090 and PIK75 and D. STA9090 and BGT226. E. Increased Caspase 3/7 activity in a dose dependent manner by the individual drugs STA9090 and PIK75 and synergistically enhanced by their combination in NCI-H295R. F. Western blot validating the decreased expression of protein markers for apoptosis (cleaved caspase-3, cleaved PARP), cell cycle proteins (cyclin B1 and CDK1), and epithelial-to-mesenchymal transition (N-cadherin and vimentin) and after combined treatment of STA9090 and PIK75 in both SW13 and NCI-H295R. G. Graph showing percentage of cells arrested in difference phases of cells cycle with a significant increase of cells arrested in the G2/M phase by STA9090 combined with PIK75 but not BGT226, as evaluated by flow cytometry. * *P*≤ 0.05, ** *P*≤ 0.01, *** *P*≤ 0.001

The anti-proliferative effect was further validated in three-dimensional (3D) multicellular aggregates (MCAs), which recapitulate *in vivo* tumor microenvironment more accurately than monolayer culture. After 4 weeks of treatments with STA9090, PIK75, BGT226 or the combination of STA9090 with either PIK75 or BGT226 at 10–40 nanomolar range, we observed higher efficacy of the combination treatments evidenced by the disintegration of SW13 and NCI-H295R MCAs (Fig. 3a, 3b). Although we found similar efficacy of the combination treatment than that of single-drug treated groups, when the MCAs were treated with the combinations of either HSP990 or NVP-AUY922 and PIK75 or BGT226 (Supplementary Fig. 8, 9), it required longer treatment duration (6–7 weeks) to induce MCA disintegration. The NVP-AUY922 combined with PIK75 with similar doses, however, did not disaggregate the MCAs effectively under the same treatment parameters. Additionally, the combination of STA9090 or HSP990 with either PIK75 or BGT226 was more effective than single-drug treatments in suppressing tumor colony formation (Supplementary Fig. 10).

Next, we assessed whether cell death was caspase-dependent apoptosis. Caspase3/7 activity in cells treated with the combinations of STA9090 or HSP9090 with PIK75 was statistically significantly higher than the control and the single drug treatment groups (Fig. 3e, Supplementary Fig. 11a, 11b, 11d). Similarly, the combination of NVP-AUY922 with PIK75 induced significantly higher apoptosis in ACC cells compared to that of the single-drug treatment and control groups (Supplementary Fig. 11b). However, statistical significance in the caspase 3/7 activity was not reached when treated with the combination of BGT226 with HSP990 (Supplementary Fig. 11c), despite cell death seen during treatment, suggesting non-apoptotic cell death by BGT226 and the respective combinations. In addition to caspase 3/7 activity, the combination of STA9090 and PIK75 induced higher expressions of cleaved caspase 3 and cleaved PARP than single-drug treated and control groups (Fig. 3f). In contrast, there was no significant increase in cleaved caspase 3 and cleaved PARP when ACC cells were treated with STA9090, BGT226, or the combination of these two drugs, suggesting that the cell death caused by BGT226 and STA9090 was not caspase or PARP-dependent apoptosis (Supplementary Fig. 12).

Because PI3K/Akt pathway is involved in cell cycle progression, the effect of the combination treatments on the status of the cell cycle was evaluated. ACC cells treated with the combination of STA9090 and PIK75 arrested G2M phase of cell cycle arrest at a higher rate compared to those in single-drug treated and control groups, with a decreased expression of G2M cell cycle regulators such as CDK1 and cyclin B1 (Fig. 3f, 3g). However, we did not observe noticeable alterations in the cell cycle when ACC cells were treated with BGT226 with or without STA9090. Thus, treatment-related cell death caused by BGT226 or the combination of BGT226 and STA9090 was not apoptotic or cell cycle-related.

Patients with ACC commonly succumb to progressive systemic metastasis, we assessed the effect of the drug combinations on migratory/invasive potential of ACC cells *in vitro*. The combinations of STA9090 and either PIK75 or BGT226 decreased the invasive and migratory potential of ACC cells (Fig. 3c, 3d and Supplementary Fig. 13) with a decrease in the expression of epithelial-to-mesenchymal (EMT) markers such as N-cadherin and vimentin (Fig. 3f). This finding suggests combination treatment was effective in reducing the metastasis compared to the individual drugs *in vitro* in ACC cells.

### Induced autophagy by combination of STA9090 and BGT226 in adrenocortical carcinoma cells

**Figure 4.**
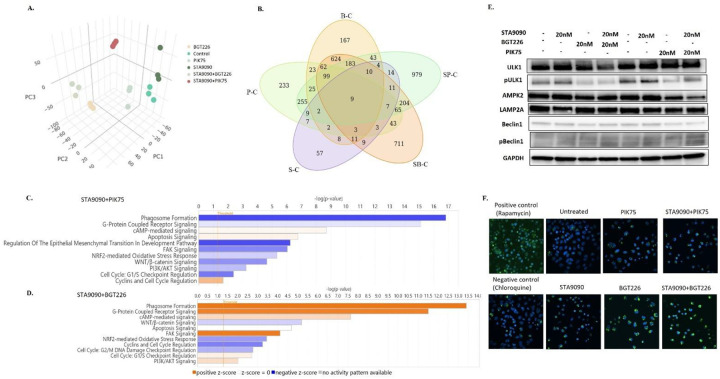
Induction of autophagy by STA9090 and BGT226. A. Principal component analysis (PCA) plot showing treatment groups distinctly segregated due to difference in their biology in NCI-H295R cells. B. Venn diagram comparing the number of genes expressed and overlapped in the comparison groups (C; Control, S; STA9090, P; PIK75, B; BGT226, SP; STA9090 and PIK75, SB; STA9090 and BGT226). Pathway analysis showing contrast in the regulation in the top canonical pathways in treatment groups C. STA9090 + PIK75 and D. STA9090 + BGT226. Each horizontal bar represents a cellular pathway, y axis: log 10 P value, x-axis: z-score, orange color for upregulation and blue color for downregulation. E. Western blots validating the protein level expression of autophagy markers in the treatment groups F. Autophagy flux analysis showing accumulation of autophagosomes consisting of LC3-GFP in the ACC cells after the treatment of drugs (STA9090 with PIK75/BGT226). Magnification; 20X, Green; GFP, Blue; DAPI nuclear stain.

Although PIK75 and BGT226 are PI3K inhibitors that target the catalytic p110α subunit of PI3K, the mechanism of ACC cell death when combined with STA9090 demonstrated differences. Hence, to characterize the difference in the mechanism of treatment-related cell death between the PIK75-STA9090 combination vs BGT226-STA9090 combination at a transcriptomic level, we carried out bulk RNA sequencing analysis to assess the differences in gene expression. In the NCI-H295R, the treatment groups clustered together in the PCA plot (Fig. 4a). Interestingly there were 711 statistically significant differentially expressed genes (DEGs) in the STA9090 and BGT226 combination compared to the control group whereas 979 DEGs exclusively expressed by STA9090 and PIK75 combination (Fig. 4b). Using the adjusted P value and the fold-change or log FC of the treatment groups, the top upregulated and downregulated genes are shown in the Volcano plots (Supplementary Fig. 14). Although PIK75 and BGT226 inhibit a common target (p110α subunit of PI3K), the gene expression profiles of ACC cells treated with these two combination regimens were quite different. Interestingly, the PI3K inhibitors caused more changes in gene expression profile of the cells than STA9090 (Fig. 4b). To further investigate the specific pathways and cellular processes involved in treatment response, we used the Ingenuity pathway analysis (IPA, Qiagen Inc., MD) and identified the upregulated or downregulated canonical signaling pathways and cellular processes. We observed distinctly different gene expression patterns between the combination of PIK75 and STA9090 compared to the combination of BGT226 and STA9090 (Fig. 4c, Fig. 4d). In contrast to the combination of PIK75 and STA9090 that induced apoptosis and downregulated autophagosome formation, the combination of BGT226 and STA9090 induced autophagosome formation (Fig. 4c and 4d). This finding was consistent with our *in vitro* data earlier showing caspase and PARP-dependent apoptosis in cells treated with PIK75 and STA9090 and the lack of changes in cell cycle, caspase 3/7 activity, expressions of cleaved caspase-3 and cleaved PARP in cells treated with the combination of BGT226 and STA9090.

To validate the treatment effect of the BGT226-STA9090 combination on autophagy, we assessed the expressions of autophagic markers in ACC cells treated with BGT226-STA9090 vs PIK75-STA9090 combination. Consistent with the pathway analysis data, there was a downregulation in the expression of phospho-ULK1 and increase in expression of phospho-Beclin1, though no significant change in AMPK2, by BGT226-STA9090 combination (Fig. 4e), whereas the change was not prominently seen in cells treated with PIK75-STA9090. We observed no change in the expression of LAMP2A suggesting that the autophagy in the BGT226-STA9090 combination was a macro-autophagy driven process due to inhibition of mTOR and not HSP90-mediated autophagy. The pattern of changes in the autophagy markers was also observed in the combination of HSP990 with either PIK75 or BGT226 (Supplementary Fig. 15). Queried mRNA expression of autophagy markers in primary human ACC samples revealed a differential upregulation of *ULK1* (P<0.01) and downregulation of *BECN1* (P<0.01), compared to normal adrenal, in two publicly available databases (GSE12368 and GSE75415, Supplementary Fig. 16), suggesting that autophagy is suppressed in these cohorts. Hence, these cohorts could possibly benefit by the drug combination treatment via induction of autophagy in the tumors. Further studies using the autophagy flux analysis demonstrated increased autophagosomes followed by treatment of either BGT226 or the combination of STA9090 and BGT226, but not by the PIK75 and STA9090 (Fig. 4f). Taken together, the combination of STA9090 and BGT226 shows induction of autophagy leading to cell death, whereas STA9090-PIK75 caused cell death through caspase-dependent apoptosis.

### ACC tumor growth was synergistically reduced by combined STA9090 and BGT226 treatment in mice xenograft model

**Figure 5.**
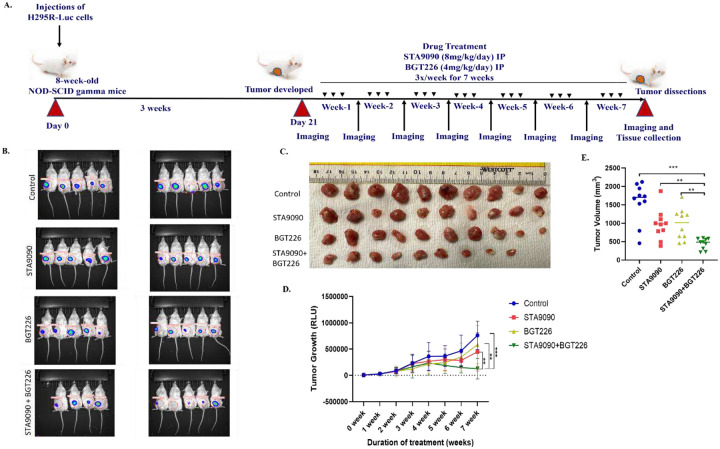
Combination therapy of STA9090 and BGT226 significantly reduced ACC tumor progression in preclinical model. A. Schematic representation of the experimental design of the mice xenograft experiment using the NOD-SCID-gamma mice. Drugs (STA9090; 8mg/kg body weight per day, and BGT226; 4mg/kg body weight per day) and were injected intraperitonially, thrice a week for 7 days, after three weeks of injecting the NCI-H295R cells with luciferase reporter. Imaging of the tumor growth were carried out at the end of the treatment every week, for 7 weeks. Images showing B. luciferase activity as captured through *in vivo* imaging system (IVIS). C. Dissected mice tumors. D. Graph represents the difference tumor growth corresponding to region of interest (ROI) intensities; combination treatment group showing significant decrease in the luciferase activity, compared to the control and individual drug treatment groups. E. Tumor measurement using the vernier calipers at the end of seventh week of the drug treatment is represented graphically. RLU, Relative light units. ** *P*≤ 0.01, *** *P*≤ 0.001

The synergistic efficacy of STA9090 and BGT226 was evaluated on ACC xenografts in immunocompromised non-obese-diabetic mice (NOD-SCID-gamma; NSG) for seven weeks (Fig. 5a). The luciferase activity of ACC xenografts in mice treated with the combination of STA9090 and BGT226 was significantly lower than that of the single-drug treatments (P<0.01) and control (P<0.001) groups after 7-weeks with a consistent antiproliferative growth curve (Fig. 5b–d). The mean volume of ACC xenografts of STA9090-BGT226 treatment group had reduced 28.4% of the vehicle control group and 38.4% and 37.7 % of those in STA9090-only and BGT226-only groups, respectively (Fig. 5e). We observed no significant change in the weight of mice or other signs of treatment-related toxicity. Of note, there was one mouse that unexpectedly died in the combination group after the 6^th^ treatment week without any signs of toxicities.

Next, we validated the *in vivo* treatment effects on PI3K pathway member phospho-AKT (ser473) and proliferation marker PCNA, using immunohistochemistry on the ACC xenograft tissues. We observed the lowest expression of phospho-AKT (ser473) in mice tissues treated with the combination of STA9090 and BGT226 compared to that of mice treated with single drugs and vehicle control (Supplementary Fig. 17).

### Patient-derived organoids respond to combined STA9090 and BGT226 synergistically

**Figure 6.**
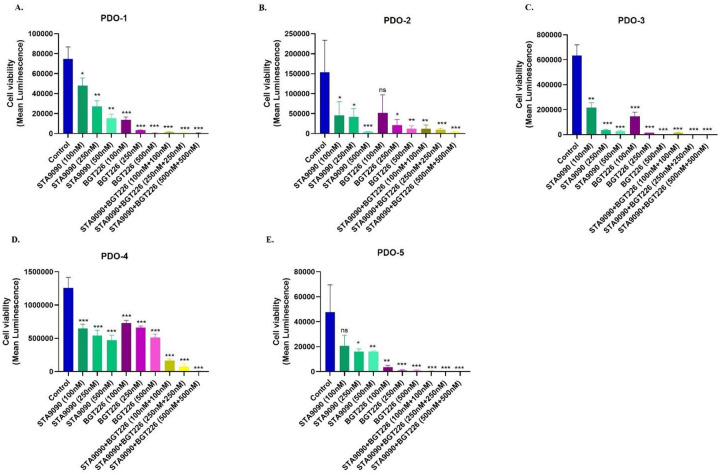
Patient derived organoids (PDOs) demonstrated significant treatment response to the drug combination treatment. A-E. Graphs representing decreased cell viability corresponding to ATP production by the PDOs, after treatment of the drugs (STA9090 and BGT226) either individually or in combination for 7 days. S, STA9090; B, BGT226; SB, STA9090+BGT226. * *P*≤ 0.05, ** *P*≤ 0.01, *** *P*≤ 0.001, ns, not significant.

Recent studies suggested that the treatment response in patient-derived organoids (PDOs) corresponded with the treatment response seen clinically^27^, thus we validated the efficacy of STA9090 and BGT226 combination in five patient-derived ACC organoids. The clinical characteristics of patient tumors are shown in (Supplementary Table. 1). In comparison to the single-drug treated and vehicle control groups, the combination of STA9090 and BGT226 showed lower viability (P<0.05), after 7 days of treatment (Fig. 6a–e). Of note, the drug concentrations that showed effective antiproliferative activity were higher than those used in the *in vitro* studies. Notably, three out of the five representative patient samples harbored pathogenic variants of *CTNNB1*. Each ACC PDO had different sensitivity to the treatments, but the combination treatment was more effective consistently across all five ACC PDOs at clinically achievable concentrations. Immunohistochemistry for SF1 was performed in the organoids to confirm ACC cells (Supplementary Fig. 18). This finding suggested that the combination of STA9090 and BGT226 may be effective in patients with advanced ACC.

## Discussion

**Figure 7.**
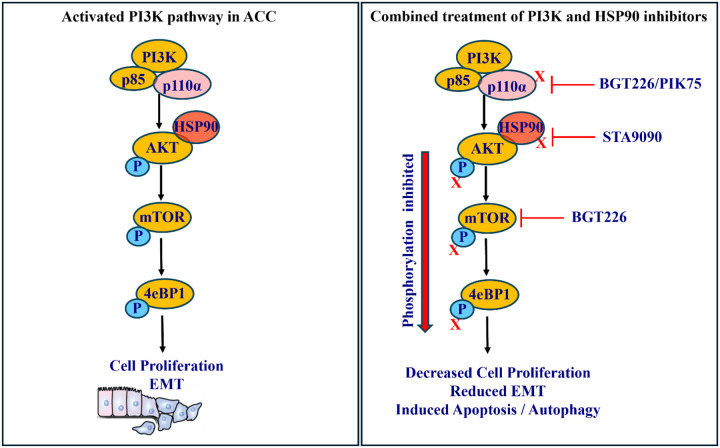
Schematic showing synergistic effects of PI3K and HSP90 inhibitors. Activation of PI3K-AKT-mTOR pathway causes cell proliferation and epithelial-to-mesenchymal transition (EMT) phenotype in adrenocortical carcinoma (ACC) cells (left panel). The BGGT226/PIK75 targets the p110 of the alpha-subunit of PI3K molecule, whereas the STA9090 targets heat shock protein 90 (HSP90), synergistically inhibiting the phosphorylation of downstream molecules of PI3K pathway, suppressing the tumorigenic processes (cell proliferation and epithelial-to-mesenchymal phenotype) and simultaneously inducing apoptosis/ autophagy in ACC (right panel).

The combinations of targeted therapy in cancers have emerged as a promising option to overcome treatment resistance. There is no effective systemic therapy for patients with advanced ACC, therefore we aimed to identify a novel combination therapy. Using qHTS we identified the combination of HSP90 and PI3K inhibitors as one of the most efficacious drug therapies in ACC and validated the efficacy *in vitro* and *in vivo* using ACC PDOs and xenografts, respectively. Our *in silico* analysis from public databases revealed overexpression of genes in the HSP90 and PI3K family and their prognostic relevance in ACC samples. We demonstrated synergistic inhibition of tumor progression and cancer hallmark processes such as proliferation, invasion/migration, and epithelial to mesenchymal transition with increased autophagy or apoptosis in ACC (Fig. 7) as a result of the combination therapy. We achieved synergy in multiple PI3K and HSP90 inhibitors suggesting the efficacy of drugs are pathway specific.

The only FDA-approved drug used for the treatment of advanced ACC is mitotane, either in combinations with cytotoxic chemotherapy or alone and demonstrated a poor response rate. Etoposide, Doxorubicin and Cisplatin (EDP) and streptozotocin are both used in the treatment of ACC^4,28^, but neither of them specifically targets the oncogenic signaling pathways that drive the tumor progression. Therefore, we focused on therapies that would target specific dysregulated molecular alterations in ACC. Among the targeted therapies, inhibitors for receptor tyrosine kinases, epithelial growth factor receptor (EGFR) and insulin-like growth factor 1 (IGF-1) receptor have shown promising efficacy in preclinical studies^29–32^. However, clinical trials failed to show clinically meaningful and durable long-term responses. Because the PI3K signaling pathway is one of the most commonly activated and dysregulated pathways in cancers and many of the kinases in this pathway are dependent on HSP90 for its stabilization^33^, the inhibition of HSP90 could be an effective therapy. We demonstrated that several members of the HSP90 (HSP90B1, HSP90B2P) and PI3K family (AKT2) were linked to shorter OS, DFS, and adverse clinical features in ACC including advanced stage and metastasis, supporting the use of the HSP90 and PI3K inhibitors in combination. HSP90 inhibitor, STA9090 demonstrated preclinical anti-tumorigenicity by disrupting the association of HSP90 with its co-chaperone p23 in *in vitro* and *in vivo* models of non-small cell lung cancer,^34^ and by degrading the key HSP90 clients such as AKT and other receptor tyrosine kinases in pheochromocytoma^35^. In addition, the inhibitor showed activity against lung adenocarcinomas driven by oncogenic ERBB2 YVMA^34^. Our data revealed HSP90 inhibition downregulated the expression of genes and proteins (phospho-AKT and phosphormTOR) involved in tumor cell survival, and proliferation, while upregulating genes related to stress responses and apoptosis.

Several second-generation synthetic HSP90 inhibitors have been studied as a monotherapy in clinical trials in patients with advanced breast cancer, gastric cancer, and non-small cell lung cancer. Ganetespib and Lumenespib in combination with chemotherapy have successfully completed phase 2/3 trials for refractory non-small cell lung cancer (NSCLC, NCT01173523)^36,37^, metastatic hormone-resistant prostate (NCT01270880)^38^, and breast cancer^39^. The safety and efficacy of STA9090 were recently tested for neoadjuvant therapy in a multicenter phase 2/3 clinical trial, I-SPY2^40^. Anti-tumorigenic activity of HSP90 inhibitor, PU-H71 was reported preclinically in Burkitt Lymphoma^33^, by targeting HSP90 clients Bcr-Abl and multiple components of PI3K/AKT/mTOR signaling pathway. In ACC, HSP90 inhibitors such as BIIB021 (B) and CCT18159 (C) were tested in the NCI-H295R cells either alone or in combination with mitotane, which revealed drug synergism with mitotane^41^. Similarly, the KU758 treatment effectively reduced migration invasion and multicellular aggregate or tumor spheroid formation along with a decrease in beta-catenin activity. Consistent with these studies our data in ACC, revealed a significant decrease in cell proliferation, spheroid formation and invasion/ migration, upon treatment with STA9090 while a modest decrease in cell proliferation, and spheroid formation by HSP990 and in AUY922. The N-terminal HSP90 inhibitor Ganetespib was reported to be more effective in decreasing cell proliferation, cell migration, and increasing apoptosis in comparison to the C-terminal inhibitors novobiocin and silibinin^42^ and can modulate ERK1/2 and AKT pathways by blocking the phosphorylation of client proteins.

Several dysregulated kinases in the PI3K/AKT/mTOR signaling pathway have been involved in multiple hallmarks of cancer such as cell proliferation and metastatic processes in multiple solid and hematologic malignancies and FDA-approved PI3K inhibitors targeting them e.g., copanlisib, idelalisib, duvelisib, umbralisib for hematologic malignancies and alpelisib for *PIK3CA* mutated advanced breast cancer^43^. One of the major challenges with PI3K inhibitors is the development of resistance. Tumors can often bypass PI3K inhibition through alternative pathways, therefore, there is a growing focus on combination therapies, to overcome resistance mechanisms. It is noted that the ability to inhibit the p110 alpha isoform is one of the most efficient mechanisms of suppressing PI3K signaling in cancers. Because PIK75, a potent selective inhibitor of p110α (with weaker affinity to p110γ) isoforms of PI3K, was identified in our qHTS finding as one of the most effective monotherapies against ACC cells, we validated *in vitro* efficacy of PIK75 and found that PIK75 effectively induced caspase-dependent apoptosis and the G2/M cell cycle arrest *in vitro*. We sought to identify another selective inhibitor of p110α isoform of PI3K that is available for a clinical trial and validate its preclinical efficacy in ACC cells. We demonstrated *in vitro* and *in vivo* efficacy of BGT226 as a monotherapy and in synergistic combination with HSP90 inhibitors. In contrast to PIK75, BGT226 (a dual inhibitor of PI3K and mTOR) induced autophagy in ACC. This was concurrent with studies that reported BGT226 induces autophagy through upregulation of microtubule-associated protein light chain 3B-II and the degradation of p62 in head and neck cancer both *in vivo* and *in vitro*^26^. Our study demonstrated combination of BGT226 with STA9090, synergistically inhibited *in vitro* and *in vivo* cell proliferation in both ACC cell lines and PDOs at clinically achievable concentrations. In addition, this combination was more effective than inhibiting clonogenicity, cell migration, and invasion. The efficacy of the combination therapy using HSP90 and PI3K inhibitors was recently reported in preclinical studies of solid tumors and lymphomas^33^, however, the combination has not been studied in clinical trials.

PI3K inhibitors target the PI3K/mTOR pathway which many cancers depend on to survive, while HSP90 inhibitors destabilize several oncogenic proteins, both leading to tumor cell death. Both drugs reduce cell proliferation, migration, and invasion in preclinical models, suggesting increased efficacy when combined. As evident from our study, the combination therapy of BGT226 and STA9090 could improve the clinical outcomes for patients with cancers harboring mutations in the PI3K/mTOR pathway. There are ongoing clinical trials investigating the combination of BGT226 (or other PI3K/mTOR inhibitors) with HSP90 inhibitors like STA9090 in other cancers, but data on safety, efficacy, and optimal dosing regimens are still emerging. Both drugs have distinct toxicity profiles, and combinations may exacerbate side effects such as gastrointestinal disturbances, fatigue, blood disorders, and potential cardiac issues. It is encouraging in our study that there were no treatment-related toxicities in mice treated with BGT226 and STA9090. In conclusion, the combination of BGT226 (a PI3K/mTOR inhibitor) and STA9090 (an HSP90 inhibitor) is effective in multiple preclinical models of ACC at clinically achievable concentrations warranting further clinical studies.

## MATERIALS AND METHODS

### Patient samples

Human adrenocortical tissue samples were collected under the clinical protocol entitled “Prospective comprehensive molecular analysis of endocrine neoplasms” (Clinical Trial Registration number NCT01005654). Ethical approval was granted by the Institutional Review Board, National Cancer Institute, NIH, and the NIH Office of Human Subject Research. All participants provided written informed consent.

### Cell culture

Human ACC cell lines, SW13 and NCI-H295R, were purchased from the American Type Culture Collection^™^ (CCL-105, CRL-2128; Manassas, VA, USA) and cultured in 5% CO_2_ atmosphere at 37 °C in Dulbecco’s Modified Eagle Medium (11195–065, Thermo Fisher Scientific, MA, USA) supplemented with 2.5% Nu-Serum (355100, Corning, MA, USA) and 0.1% Insulin-Transferrin-Selenium (41400045, Thermo Fisher Scientific, MA, USA). Cell lines were authenticated by short tandem repeat profiling. See supplementary methods and materials for details.

### Small molecule inhibitors and *in vitro* treatments

Small molecule inhibitors Ganetespib (STA-9090; S1159), Luminespib (NVP-AUY922; S1069), HSP990 (NVP-HSP990; S7097), PIK-75 (PIK-75 HCl; S1205) and BGT226 maleate (NVP-BGT226; S2749) were purchased from Selleck Chemicals LLC (Houston, TX, USA). See supplementary methods and materials for details.

### Quantitative high-throughput drug screening and combination matrix analysis

The National Center for Advancing Translational Sciences (NCATS) Pharmaceutical Collection (NPC) and the Mechanism Interrogation PlatE (MIPE) library, which in total consisted of 4,991 approved and investigational compounds, were primarily screened against SW13 and NCI-H295R cell lines using their cell viability measured by CellTiter-Glo^®^ (Promega, Madison, WI), as described in our previous studies^20,21^. See supplementary methods and materials for details.

### Gene expression profiling

Publicly available genome-wide mRNA expression data were downloaded and analyzed from three Gene Expression Omnibus (GEO) cohorts (GSE10927, GSE12368, and GSE75415) using embedded interactive statistical software (GEO2R). The relative mRNA expressions of genes of interest in ACC samples were compared to those of adrenocortical adenoma (ACA) and/or normal adrenal tissue samples. The *p* values were adjusted for a false discovery rate using Benjamini-Hochberg method.

We obtained and analyzed transcriptomic and relevant clinical data of primary human ACC samples from the Cancer Genomics Atlas (TCGA) database for Adrenocortical Carcinoma (total samples, N=79) from C-Bioportal (http://www.cbioportal.org), hosted by the Computational Biology Center at Memorial-Sloan-Kettering Cancer Center for Cancer Genomics. Query was performed for somatic mutations, copy number alterations, mRNA expression, and protein expression of the key genes involved in the regulatory functions of HSP90 (HSP90AA1, HSP90AB1, HSP90B1, TRAP1), AKT2, PIK3CA, and CDK1 and percentage alterations of each gene in the database and mutual exclusivity or co-occurrence was evaluated.

### Cell Proliferation Assay

ACC cells NCI-H295R (n=6000 cells/well) and SW-13 (n=2000 cells/well)) were seeded in 96-well plates and 48 hours later were treated in triplicates with a series of concentrations of either vehicle (DMSO), HSP90 inhibitor (STA-9090, HSP990 or NVP-AUY922), and PI3K inhibitor (PIK-75 or BGT226) individually or in combinations for 7 days. The antiproliferative activity was evaluated using the CyQuant^®^ Cell Proliferation Assay (Invitrogen^™^ Corp., Carlsbad, CA) as per the manufacturer’s protocol, quantified by SpectraMax i3× 96-well fluorescence plate reader (Molecular Devices, Sunnyvale, CA) at 485nm/538nm. See supplementary methods and materials for details.

### Clonogenic Assay

ACC cells were seeded in triplicate in 6-well plates (1000 cells/well) and allowed to grow for 1 week followed by treatment with drug(s) alone or in combination or with the vehicle in complete media. Growth media with vehicle or drug(s) were replaced twice every week. Treated or untreated cells were allowed to form colonies for another 3 weeks, and then fixed with 0.4% buffered paraformaldehyde and then stained with 0.5% crystal violet prepared in 25% methanol for 10 min. The colonies were counted and photographed using a ChemiDoc system (Bio-Rad Laboratories, Hercules, CA) and quantified manually for the number of colonies.

### Three-dimensional multicellular aggregates (MCA)

To create 3-dimensional multicellular aggregates (MCA), SW13 or NCI-H295R (1 × 10^5^ cells/0.5 mL) cells were seeded in an Ultra-Low Cluster, 24-well plate (Costar catalog #, Corning, NY), incubated at 37°C with 5% CO2 for two weeks. Next, we treated these MCAs with monotherapy and in combination using the above inhibitors for 3 weeks. The MCAs or tumor spheroids were treated continuously with intermittent change of growth media with or without inhibitors. Spheroids were observed and images were captured under the light microscope (magnification 40x) and compared for their sizes and integrity.

### Apoptosis Assay

The caspase 3/7 activity was estimated to quantify the apoptotic potential and was evaluated using the Caspase-Glo 3/7 Assay kit (G8091, Promega North America, Madison, WI) according to the manufacturer’s protocol. Briefly, SW13 (6 × 10^3^ cells/well in 100 μl) and NCI-H295R (6 × 10^3^ cells/well in 100 μl) were seeded in a 96-well white-walled clear bottom plate (Lonza, Allendale, NJ) were incubated for 24 hours at 37°C in 5% CO2 and then treated with the standardized concentrations of inhibitors or respective vehicle. After 48 and 72 hours of treatment, 100 μl of the Caspase-Glo 3/7 reagent was added to each well including blank wells containing only culture medium, vehicle control, and treated cells in culture medium, contents were gently mixed using a plate shaker for 30 seconds. Plates were covered with aluminum foil and incubated at room temperature for 30 minutes. Luminescence was measured by the SpectraMax i3Max plate reader (Molecular Devices, Sunnyvale, CA) per the manufacturer’s protocol.

### Invasion/migration Assay

We assessed cellular migration and invasion by using the Corning Biocoat cell culture 24-multiwell insert system (354578, 354480, Corning, Glendale, AZ) according to the manufacturer’s protocol. See supplementary methods and materials for details.

### Western Blotting

Total protein was extracted from cells (SW13 and NCI-H295R, treated or untreated with either STA9090 or PIK75/BGT226 or both for 48 hrs and 72hrs) using RIPA lysis buffer (ThermoFischer Scientific, MA) with Halt protease inhibitor (ThermoFischer Scientific, MA), and protein concentration was determined using Bradford assay. An equal amount of protein concentration (50μg) from each treatment condition was resolved by electrophoresis using SDS-PAGE in 4–20% tris-glycine gels. Protein bands were analyzed using an enhanced chemiluminescence (ECL) reagent (Pierce, ThermoFisher Scientific, MA), and images were captured in ChemiDoc^™^ MP Imaging System (Bio-Rad Laboratories, Hercules, CA), according to the manufacturer’s instructions. See supplementary methods and materials for details.

### Cell cycle analysis

Cells (SW13 and NCI-H295R) treated or untreated with either STA9090 or PIK75/BGT226 or both for 48 hrs) were trypsinized, counted (1 × 10^6^ cells/500 μl for analysis), washed twice with phosphate-buffered saline (PBS), fixed with ice-cold 70% ethanol and stained with FxCycle^™^ Propidium Iodide/RNase Staining Solution (Thermofisher Scientific, MA) followed by quantification of stained cells by flow cytometry analysis using the BD Fortessa flow cytometer (Becton Dickinson, Franklin Lakes, NJ, USA). Data were generated for at least 20,000 events per sample. The cell cycle of the gated PI distribution was analyzed using FlowJo software (Becton Dickinson and Company, USA).

### RNA sequencing analysis

Gene expression profiling was performed using bulk RNA sequencing in ACC cells (NCI-H295R and SW-13) treated with 20nM of HSP90 or PI3K inhibitors alone or in combinations, for 48 hours, followed by mRNA extraction (RNeasy Mini Kit, 74104, Thermofisher Scientific) and quantification by Nanodrop 1000 (Thermofisher Scientific). The integrity of isolated RNA was evaluated with the Agilent 2100 Bioanalyzer (Agilent Technologies). RNA sequencing was performed at the Sequencing Facility, Leidos Biomedical Research, Inc., Frederick National Laboratory for Cancer Research Sequencing. See supplementary methods and materials for details.

### Pathway analysis

The Ingenuity Pathway Analysis (IPA, Qiagen Inc.) was used to identify significantly upregulated or downregulated signaling pathways in each of the treatment groups. See supplementary methods and materials for details.

### Autophagy flux analysis

Autophagic activity in the live ACC cells was evaluated using an Autophagy detection kit (ab139484, Abcam) as per the manufacturer’s protocol. See supplementary methods and materials for details.

### *In vivo* study with human ACC xenografts

The protocol designed to study the *in vivo* efficacy of the combination of STA9090 and BGT226 in mice with human ACC xenografts was approved by the Animal Care and Use Committee, National Cancer Institute, National Institutes of Health (NIH). Mice were maintained according to NIH Animal Research Advisory Committee guidelines. NCI-H295R cells with luciferase reporter (5×10^6^) prepared in 100μl Matrigel (Corning^®^ Matrigel^®^ Matrix (Cat # 354234, Corning, NY, USA) and serum-free growth media (50:50) were subcutaneously injected into the unilateral flank of 8-weeks old non-obese diabetic/severe combined immunodeficiency mice (NOD/SCID gamma, NOD.Cg-*Prkdc*^*scid*^ *Il2rg*^*tm1Wjl*^/SzJ, Jackson Laboratory, Bar Harbor, ME) to form ACC xenografts. Five mice were housed per cage and given regular food and water ad libitum. The tumor burden of ACC xenografts was monitored weekly using the Xenogen IVIS Spectrum *in vivo* imaging system (PerkinElmer, Shelton, CT, USA). See supplementary methods and materials for details.

### Patient-derived Organoids

Following surgical resection, tumor tissues were washed twice with 1% Penicillin/Streptomycin in PBS anti-biotic solution (15070063, Gibco). Tissues were dissected to remove necrotic or non-cancerous regions then minced into 1–2 mm pieces, transferred into tubes containing tumor dissociation kit (130-095-929, Miltenyi Biotec, MD, USA) and homogenized using a gentle MACS Octo Dissociator with Heaters (130-096-427, Miltenyi Biotec) according to manufacturer’s instructions. See supplementary methods and materials for details.

### Immunohistochemistry

Human ACC or adrenocortical adenoma (ACA) tissue samples and PDOs were formalin-fixed, embedded in paraffin, and 5-μm-thick sections were used for hematoxylin and eosin (H&E) staining. Immunohistochemistry (IHC) analysis was performed for the targeted proteins using their primary antibodies. See supplementary methods and materials for details.

### Statistical analysis

Gene expression profiling data from GEO databases were analyzed using embedded interactive statistical software (GEO2R). The *p* values were adjusted for the false discovery rate using the Benjamini-Hochberg method. For the *in vitro* experiments, and the associations between mRNA expression and clinical features, the student’s t-test was used to compare the mean between groups that were normally distributed, or the Mann–Whitney *U* test was used to compare continuous variables with non-parametric distribution. A two-tailed *p*-value less than 0.05 was considered statistically significant. Analysis of variance (ANOVA) with post-hoc tests to compare the mRNA expression and *in vivo* luciferase activity between treatment groups. We used Pearson’s and Spearman’s correlation to assess the correlations between continuous variables such as mRNA expression data in TCGA cohort with parametric and non-parametric distribution, respectively. We compared the estimated overall survival (OS) and disease-free survival (DFS) of patients with primary ACC in TCGA cohort using the Kaplan-Meier method with a log-rank test in a dichotomized cohort using the median as a cutoff for mRNA expression of the selected genes. All the statistical analyses were performed using GraphPad Prism 10 software (GraphPad Software, La Jolla, CA, USA) and the IBM SPSS Statistics 29.0 (IBM, Inc, Amork, NY).

## Supplementary Files

This is a list of supplementary files associated with this preprint. Click to download.

- SupplementaryinformationMaterialsandMethods.pdf
- westernfullscansformanuscript.pdf
- Supplementaryfigures.pdf

## Figures and Tables

**Table 1:** Correlations between mRNA expression of *HSP90* family members with *MKI67* and *CTNNB1* in human ACC samples from the TCGA cohort.

Genes	Spearman Correlation Coefficient Value	Corresponding “*p*” Value
*HSP90B3P vs MKI67*	0.40	P = 2.422e–4
*HSP90B2P vs MKI67*	0.47	P = 1.280e–5
*HSP90B1 vs MIK67*	0.48	P = 7.0546–6
*HSP90B2P vs CTNNB1*	0.55	P = 1.42e–7
*HSP90AB3P vs MKI67*	0.25	P = 0.0243
*HSP90AB2P vs MKI67*	0.28	P = 0.0132

*P* values were evaluated by t-test
